# Context-specific protection of TGFα null mice from osteoarthritis

**DOI:** 10.1038/srep30434

**Published:** 2016-07-26

**Authors:** Shirine E. Usmani, Veronica Ulici, Michael A. Pest, Tracy L. Hill, Ian D. Welch, Frank Beier

**Affiliations:** 1 Department of Physiology & Pharmacology, Schulich School of Medicine & Dentistry, The University of Western Ontario, London, ON, Canada; 2 Department of Animal Care and Veterinary Services, University of Western Ontario, London, Canada

## Abstract

Transforming growth factor alpha (TGFα) is a growth factor involved in osteoarthritis (OA). TGFα induces an OA-like phenotype in articular chondrocytes, by inhibiting matrix synthesis and promoting catabolic factor expression. To better understand TGFα’s potential as a therapeutic target, we employed two *in vivo* OA models: (1) post-traumatic and (2) aging related OA. Ten-week old and six-month old male *Tgfa* null mice and their heterozygous (control) littermates underwent destabilization of the medial meniscus (DMM) surgery. Disease progression was assessed histologically using the Osteoarthritis Research Society International (OARSI) scoring system. As well, spontaneous disease progression was analyzed in eighteen-month-old *Tgfa* null and heterozygous mice. Ten-week old *Tgfa* null mice were protected from OA progression at both seven and fourteen weeks post-surgery. No protection was seen however in six-month old null mice after DMM surgery, and no differences were observed between genotypes in the aging model. Thus, young *Tgfa* null mice are protected from OA progression in the DMM model, while older mice are not. In addition, *Tgfa* null mice are equally susceptible to spontaneous OA development during aging. Thus, TGFα might be a valuable therapeutic target in some post-traumatic forms of OA, however its role in idiopathic disease is less clear.

Osteoarthritis (OA) is the most prevalent joint disease and the most common cause of physical disability in western society. Patients with OA experience joint pain and impaired function that will ultimately affect their ability to work and their quality of life^1^,^2^. Currently there are no treatment options that prevent, cure, or alter the disease course. Rather, therapeutic options are limited to symptom management^3^. The financial consequences of managing OA are immense and relate to symptomatic treatment, joint replacement surgeries, and work lost to disability^4^,^5^. Current estimates suggest that developed nations spend approximately one to two percent of their gross domestic product on OA and other rheumatic diseases^6^. Furthermore, due to high rates of obesity and the aging population, the incidence of OA is only expected to rise in the coming years^7^. Consequently the identification of good therapeutic targets for the development of disease modifying osteoarthritic drugs (DMOADs) is urgently needed.

The defining feature of OA is the degeneration of articular cartilage – the tissue that covers the ends of diarthrodial joints and enables their smooth, wear-resistant movements. Articular cartilage consists of an extracellular matrix rich in type II collagen and proteoglycans and is designed to absorb and disperse the forces experienced during joint loading^8^. The extracellular matrix is sparsely populated by chondrocytes that are responsible for synthesizing the matrix and regulating its turnover^9^. A number of growth factors, cytokines, and catabolic enzymes are involved in normal cartilage metabolism as well as in OA^10^. As of yet however, no drugs aimed at these therapeutic targets have been successful in preventing, stopping or reversing the disease process in clinical trials^11^.

While the exact etiology of OA remains unclear, numerous risk factors have been identified^12^,^13^. Two of the most significant are joint injury and advanced age. It has been well established that a history of joint injury is a major risk factor for the development of OA^14^. Causes of post-traumatic OA include intra-articular fractures, ligament tears, meniscal injuries, and cartilage micro-trauma^15^. In the United States, meniscal injuries represent the highest proportion of all intra-articular injuries^16^. Menisci help nourish and lubricate the articular cartilage, act as shock absorbers, and transmit over half of the total axial load applied to the knee joint^16^. Studies have indicated that individuals with ligamentous or meniscal injuries increase their risk of developing OA by ten-fold^15^,^17^.

In addition to injury, advancing age is another major risk factor for OA. It is unusual to find individuals over the age of sixty-five to seventy without radiographic evidence of OA^18^. Several theories have been developed to describe the link between aging and OA, although there is still a lack of consensus in this area. Perhaps the oldest theory is that OA is a “wear and tear” disease brought about by repetitive loading and an accumulation of damage over time^19^, but this does not explain why radiographic signs are completely absent in some elderly individuals^20^,^21^. Other theories relate to specific age-related changes observed in the articular cartilage matrix and chondrocytes themselves. For example, aged cartilage undergoes more collagen cross-linking, thus making it stiffer and perhaps more susceptible to mechanical injury^22^. As well, decreases in aggrecan deposition are seen with age, altering the cartilage’s ability to deal with compressive forces^19^. Aging also brings about various changes within the chondrocytes themselves including decreased responsiveness to anabolic cytokines, and increased generation of reactive oxygen species (ROS)^18^. Finally, remaining theories relate to aging and chondrocyte senescence, autophagy, and apoptosis^18^,^19^,^22^.

In order to better understand the molecular mechanisms involved in OA, our lab recently studied a rat anterior cruciate ligament transection/partial meniscectomy (ACLT/PM) model to identify genes involved in cartilage degeneration^23^,^24^. We identified transforming growth factor alpha (TGFα) as a novel growth factor potentially involved in OA as its expression was increased in the disease state. Subsequent *in vitro* experiments showed that treatment of rat primary chondrocytes with recombinant TGFα resulted in a loss of chondrocyte phenotype; we observed decreases in anabolic factors including type II collagen and aggrecan and increases in catabolic factors such as matrix metalloproteinase 13 (MMP13)^25^. These effects were mediated by a number of intracellular pathways including the MEK/ERK and Rho/ROCK pathways^26^. In this study, we wanted to determine the role of TGFα in the progression of OA *in vivo* and thus employed a post-traumatic OA model in the *Tgfa* knockout mouse. The OA model we used was the destabilization of medial meniscus (DMM) model, which provides a relatively slowly progressing form of the disease that may have more relevance to the human disease course^27^,^28^. We also observed spontaneous development of OA in aging *Tgfa* null mice. Since our previous studies suggested an overall catabolic effect of TGFα on chondrocytes, we hypothesized that *Tgfa* null mice would experience delayed OA progression in both the DMM model and in spontaneous disease development.

## Results

### Young adult *Tgfa* null mice experience delayed OA progression in a DMM model

**Figure 1:**
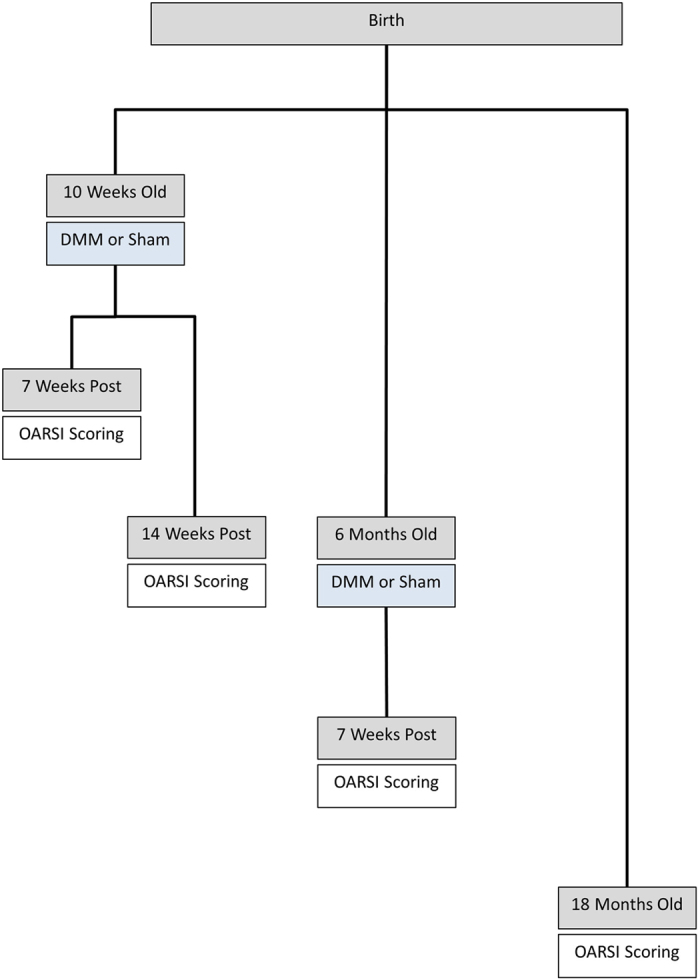
A schematic representation of the post-traumatic and spontaneous aging models of OA used in this study. Ten-week old male *Tgfa* null and heterozygous mice underwent destabilization of medial meniscus (DMM) surgery. Two post-surgical time points were assessed with OARSI scoring: seven and fourteen weeks. Six-month-old *Tgfa* null and heterozygous mice also underwent DMM surgery and were assessed seven weeks post-surgery. Lastly, *Tgfa* null and heterozygous mice were housed for eighteen months with no surgical intervention, after which time spontaneous disease progression was assessed with OARSI scoring.

**Figure 2:**
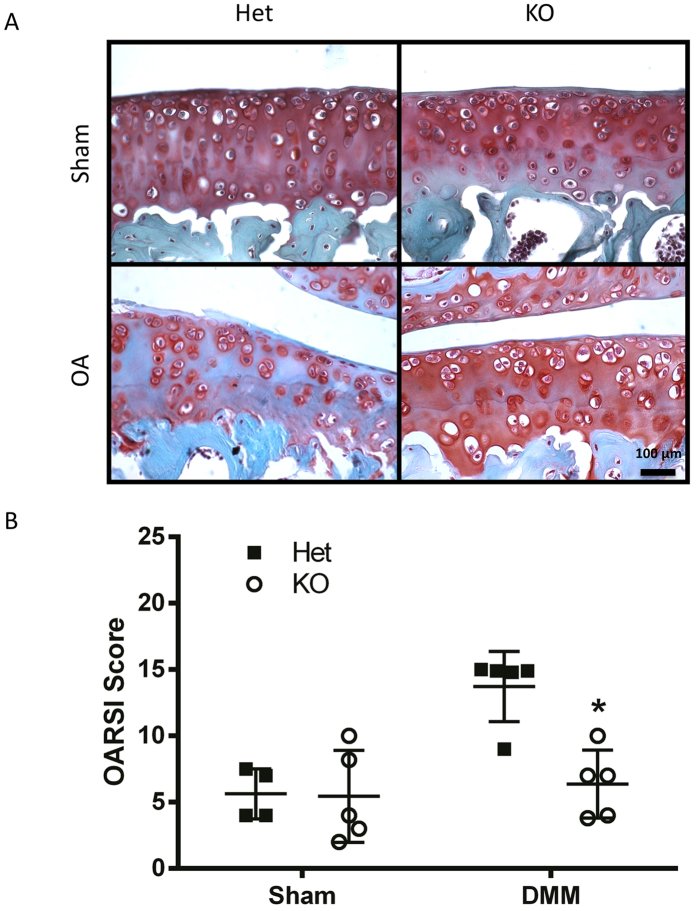
*Tgfa* knockout mice have lower OARSI scores than control littermates in a surgical model of OA. Ten-week-old *Tgfa* knockout mice and their heterozygous littermates received either surgery resulting in the destabilization of the medial meniscus (DMM) or sham surgery. At seven weeks post surgery, mice were sacrificed and their knee joints were prepared for histology. Tissues were stained with safranin-O and fast green (**A**) and then scored using the OARSI system for OA histopathology (**B**). Independent data points for OARSI scores with mean ± SD are shown and indicate that *Tgfa* knockout mice have lower scores than OA heterozygous mice after DMM surgery (DMM groups n = 5, sham groups n = 4, *p < 0.05).

To examine the effects of *Tgfa* deficiency on post-traumatic OA in young adult mice, male 10 week-old heterozygote or homozygote mutant mice underwent destabilization of the medial meniscus (DMM) or sham surgery as depicted in Fig. 1. All sham animals retained relatively healthy cartilage with Osteoarthritis Research Society International histopathology (OARSI) scores below 6.0 (Fig. 2A,B). Many disease features were apparent at the 7 week post-surgery time point in heterozygote (control) mice of the DMM group, including chondrocyte clustering, hypertrophy, loss of proteoglycan staining in the matrix, and superficial zone fibrillation (Fig. 2A). This indicated that DMM induced cartilage degeneration resembling OA pathology, as expected. More severe disease features included superficial zone delamination and lesions extending into the midzone (Fig. 2A). At seven weeks post-surgery, the average OARSI score for heterozygous DMM mice was 13.7, while the score in the sham surgery group was 5.6 (Fig. 2B). *Tgfa* knockout mice that received the DMM surgery showed significantly lower levels of cartilage damage and OARSI scores (average of 6.4) than their heterozygous littermates, and had similar scores to sham-operated heterozygote and homozygote mice (Fig. 2B).

**Figure 3:**
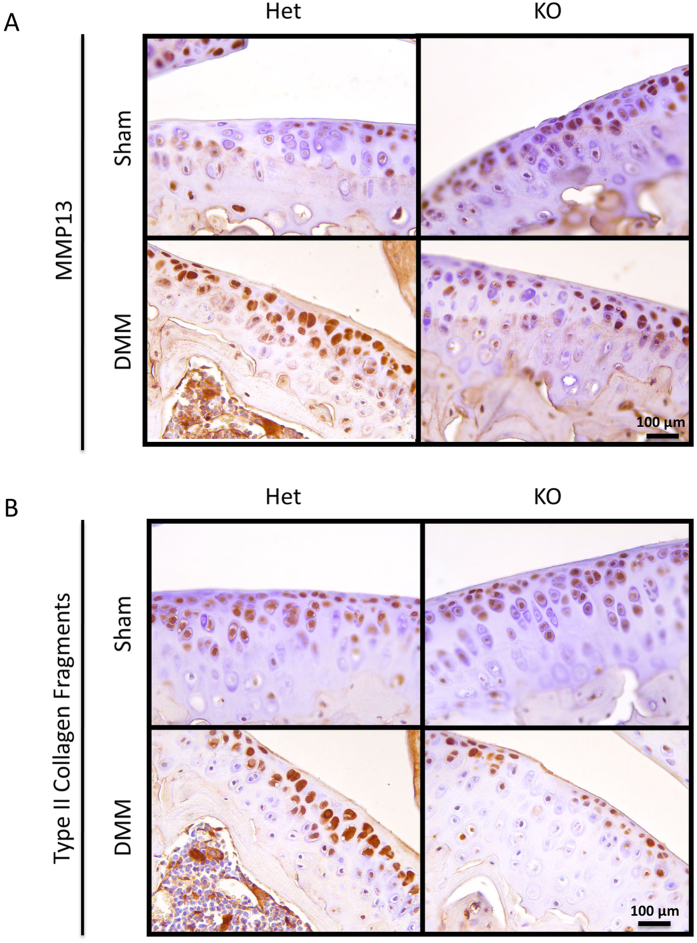
*Tgfa* knockout mice express less MMP13 and less type II collagen neoepitopes than control littermates at 7 weeks post-surgery. Histological sections were prepared from the knee joints of both *Tgfa* knockout and heterozygous mice at seven weeks post-surgery. Immunohistochemistry was performed with primary antibodies against MMP13 and type II collagen fragments. This was followed by secondary antibody incubation and visualization with the substrate DAB (brown precipitate). Nuclei were counterstained with hematoxylin (blue). Representative images show that knockout animals express less MMP13 and type II collagen fragments than heterozygous controls.

We also examined molecular markers by immunohistochemistry to further validate our histological findings. Representative images show higher MMP13 staining in the articular cartilage of heterozygous DMM animals than in knockout DMM animals or in sham animals of either genotype (Fig. 3A). Similarly, there appeared to be stronger type II collagen neoepitope staining in the cartilage of heterozygous DMM animals than in that of their knockout littermates or sham controls (Fig. 3B). Interestingly, when we evaluated MMP3 by immunostaining, no observable differences were identified between any groups (Supplementary Figure 1).

**Figure 4:**
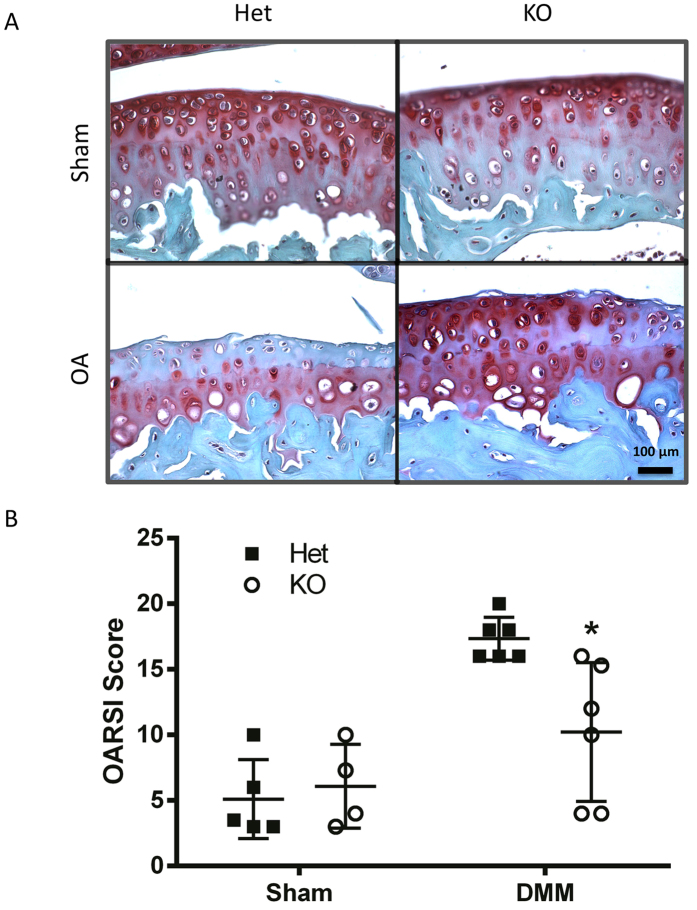
*Tgfa* knockout mice still show resistance to developing OA at 14 weeks post-surgery. 10 week-old *Tgfa* knockout mice and their heterozygous littermates received DMM surgery or sham surgery. At fourteen weeks post surgery, mice were sacrificed and their knee joints were prepared for histology. Tissues were stained with safranin-O and fast green (**A**) and then scored using the OARSI system for OA histopathology (**B**). DMM knockout animals still displayed lower OARSI scores than their heterozygous control littermates at this time point (DMM groups n = 6, sham groups n = 4, *p < 0.05). Independent data points for OARSI scores with mean ± SD are shown.

Similar results were seen when mice were analyzed fourteen weeks after surgery; heterozygous DMM animals had significantly higher scores (17.3) than knockout DMM animals (10.2) (Fig. 4B). Many more heterozygote DMM animals displayed advanced disease features such as superficial zone delamination and lesions extending into the midzone (Fig. 4A), and displayed lesions extending to the calcified cartilage. Scores for both DMM groups were higher than at the seven week time point, indicating that the disease had progressed. Both sham groups had similar scores and showed some loss of proteoglycan staining (Fig. 4B).

### 6-month-old *Tgfa* null mice are not protected from OA progression in a DMM model

**Figure 5:**
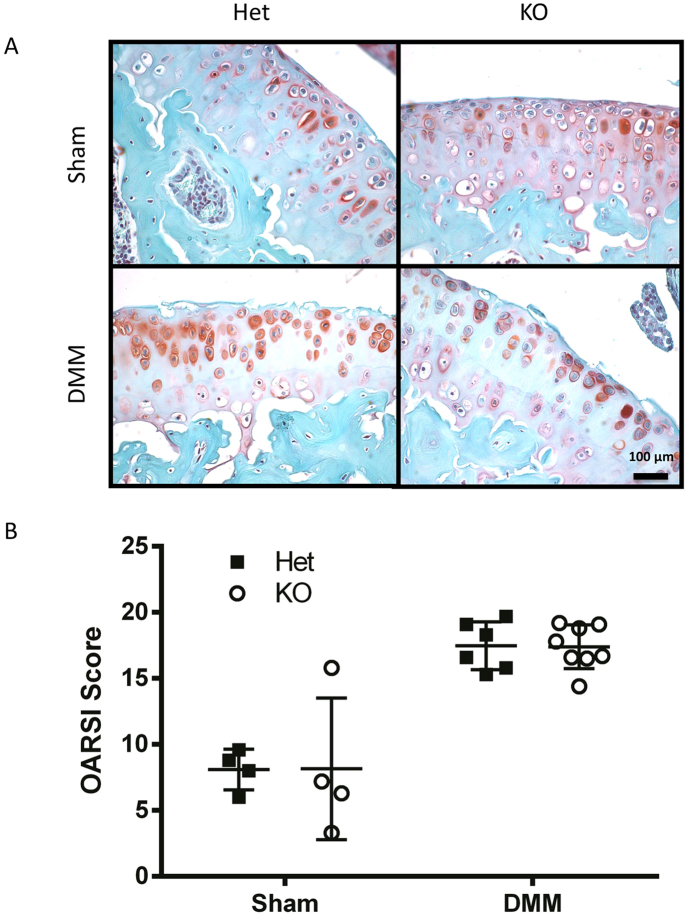
There is no difference in disease progression between knockout and control animals after DMM surgery in middle-aged mice. Six-month-old *Tgfa* knockout and heterozygous mice received DMM or sham surgery. At seven weeks post surgery, these mice were sacrificed and their knee joints were isolated and prepared for OARSI scoring. Independent data points for OARSI scores with mean ± SD are shown and indicate that DMM knockout and heterozygous mice are equally susceptible to developing OA in this model (sham groups n = 4, DMM Het n = 6, DMM KO n = 8, *p < 0.05).

These results suggested that loss of TGFα signaling protects from post-traumatic OA in young adults, a finding of significant impact for the human population because of the high number of sports-related injuries in this age group. However, human joint injuries can also occur at later ages, thus combining two OA risk factors (aging and injury). To address this point, we performed DMM and sham surgery on six-month-old mice. OA progression was more advanced than when surgery was performed on younger mice (Fig. 5A). Both *Tgfa* knockout mice and their heterozygous littermates had average OARSI scores of 17.1 when assessed at seven weeks post-surgery (Fig. 5B). A number of disease features were evident including proteoglycan loss, superficial zone delamination and erosion of midzone cartilage (Fig. 5A). In addition to the disease features mentioned above, some six moth old tissues revealed denudation to the calcified zone. *Tgfa* null mice were no longer protected from developing OA, since the knockout mice had scores and histopathological features similar to those of the heterozygous mice (Fig. 5B).

### *Tgfa* null mice are not protected from spontaneous OA

**Figure 6:**
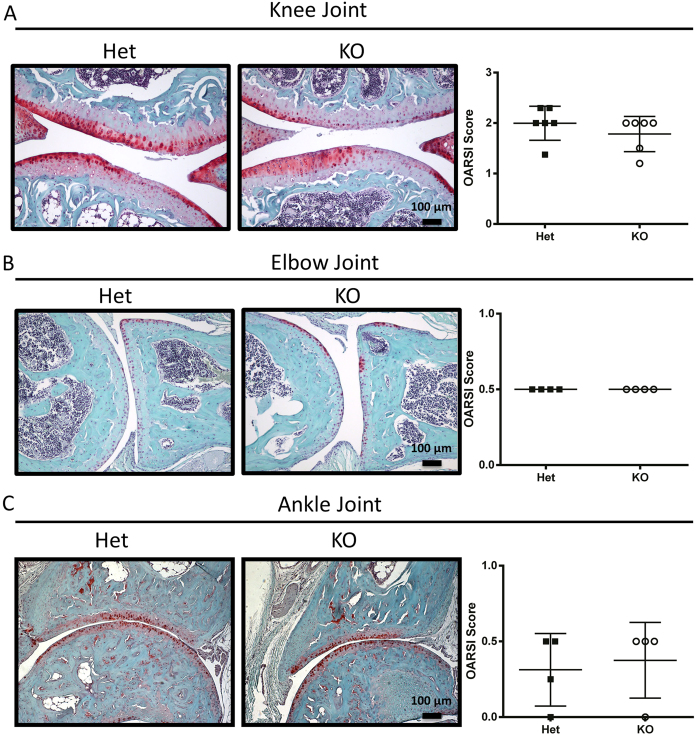
*Tgfa* knockout and heterozygous mice are equally susceptible to developing OA during aging. Male *Tgfa* knockout and heterozygous mice were housed until eighteen months of age. Animals were then sacrificed, and their knee (**A**), elbow (**B**) and ankle (**C**) joints were prepared for histology. Tissues were stained with safranin-O and fast green and scored using the OARSI recommendations for histopathological assessment of OA in the mouse. There were no differences in OARSI scores between genotypes for any of the joints examined (knee n = 7, elbow n = 4, ankle n = 4, p < 0.05). Independent data points for OARSI scores with mean ± SD are shown.

To address a potential role of TGFα in aging-associated OA, the knee, elbow, and ankle joints of eighteen-month-old mice were examined for spontaneous OA. Female mice of both genotypes were essentially completely protected from developing OA (data not shown). This finding has been well established in the literature and is attributed to differences in sex hormones^29^. The knee joints of male mice showed the most advanced disease of the joints examined, revealing areas of proteoglycan loss, some areas of superficial zone delamination, and some loss of the midzone cartilage. Regardless of the genotype, however, articular cartilage remained largely intact, and areas of cartilage loss were focal in nature (Fig. 6A). No differences between genotypes were observed. The elbow and the ankle cartilage for both *Tgfa* null and heterozygous mice was relatively healthy and showed only initial signs of OA, particularly proteoglycan loss (Fig. 6B,C). Essentially all of the tissues examined from the elbow and the ankle had an intact articular cartilage surface, free of fibrillations and fissures (Fig. 6B,C).

## Discussion

Previously, our lab identified TGFα as a novel growth factor involved in the degeneration of articular cartilage^23^,^25^. In this study, we examined the role of TGFα in the progression of OA *in vivo* through the use of the *Tgfa* null mouse. We analyzed two separate models of OA: the post-traumatic DMM model as well as a model of OA in aging mice. When young adult, ten-week old *Tgfa* null mice received DMM surgery, they were protected from developing OA at both post-surgical time points examined. While they still showed signs of degeneration, particularly at the later time point (14 weeks after surgery), disease progression was histologically less severe than that of control littermates. These data demonstrate that in the absence of TGFα mice are protected from OA development in a post-traumatic model, consistent with our previous *in vitro* experiments that showed TGFα induced an OA-like phenotype in articular chondrocytes^25^. Furthermore, we have very recently shown that pharmacologic inhibition of the receptor for TGFα, EGFR, reduces OA progression in post-traumatic rat model of OA^30^. Thus by eliminating TGFα at the genomic level, we expected that we would see similar protective effects.

Contrary to our hypothesis however, the *Tgfa* null mice were not protected from developing OA when compared to controls in our aging model or when DMM was performed in older mice. Several changes occur in aging cartilage that could potentially make it more susceptible to injury, such as a more heavily cross-linked extracellular matrix, a loss in aggrecan content, and chondrocytes that are less responsive to anabolic factors^19^. Furthermore, studies have shown that in the C56 BL/6 mouse articular cartilage thickness decreases with age, chondrocyte death increases, and the overall number of chondrocytes decreases as well^31^. These changes within the tissue provide one potential explanation for why our older *Tgfa* null mice were indistinguishable from their control littermates with regards to OA progression. In addition to the articular cartilage itself, changes within the broader context of the joint are seen with aging. A recent study by Loeser *et al*. examined the differences in gene expression within the entire joint in young and old mice receiving either sham or DMM surgery^32^. In this particular study, twelve week old mice were used for the young group and twelve month old mice were used for the aged group^32^. Interestingly, many genes were found to be dysregulated between the young and aged groups post DMM surgery, as well as between the young and old sham-operated mice^32^. This study clearly illustrates baseline differences in gene expression between joint tissues of young and old mice as well as differences in gene expression changes in response to joint injury^32^.

Our data suggest that TGFα and its downstream signaling pathway might be a good therapeutic target for OA therapy. TGFα is a member of the epidermal growth factor (EGF) family and signals through the EGF receptor (EGFR)^33^,^34^,^35^. Recent studies have shown that mutant mice with enhanced EGFR signaling develop early, spontaneous degenerative disease in multiple joints the including knee, ankle, and temporomandibular joints^36^,^37^. These mice have a mutation in mitogen-inducible gene 6 (MIG6), also known as RALT or Gene 33^36^. MIG6 is a cytoplasmic protein that negatively regulates EGFR signaling through several mechanisms, including inhibition of the receptor kinase domains and receptor internalization and degradation^38^,^39^,^40^,^41^. These mutant mice have classical arthritic features such as loss of proteoglycan content, degradation of articular cartilage, formation of subchondral cysts, synovial hyperplasia, osteophyte formation, and abnormal calcification^36^,^37^. Moreover, *Mig6* knockout mice (that have increased EGFR signaling) that underwent surgical models of OA have accelerated and increased joint damage compared to controls^42^. Furthermore, cartilage-specific deletion of *Mig6* results in an OA-like phenotype including chondrocyte proliferation and subchondral bone changes, as well as erosion of ligament insertion sites and the formation of osteophyte-like nodules^43^,^44^.

However, these cartilage-specific *Mig6* KO mice also revealed a more complex phenotype where loss of MIG6 and subsequent activation of EGFR signaling has an early anabolic effect on cartilage. This anabolic phenotype is transient, suggesting a biphasic or context-specific role of EGFR signaling in articular cartilage. Such a context-dependent role is also supported by a recent study showing that EGFR inhibition protects from OA in a mouse model^45^, which is opposite to the data presented here. It seems highly likely that factors such as extent of EGFR inhibition/activation, the timing of these manipulations, and the severity of the disease can affect whether EGFR signaling is protective or destructive.

One difference between the MIG6 studies and our own is that signaling from all EGFR ligands is inhibited while we specifically targeted TGFα. Thus, compensation by other EGF family members is another possible explanation for the lack of protection from OA in aging *Tgfa* null mice. For example, a recent study has shown increased expression of HB-EGF in osteoarthritis^46^. As well, we have recently described a transient developmental bone phenotype in our *Tgfa* null mice that appears to resolve by ten weeks of age (the age that DMM surgeries are performed in this study)^47^. However, we cannot be certain that the effects of this developmental phenotype, which include delayed osteoclast recruitment and decreased MMP13 and RANKL (receptor activator of nuclear factor kappa B ligand) gene expression, do not have an effect on the outcome of our OA studies^47^. However, the fact that sham-operated controls and aging mice showed no differences between genotypes suggests that any such developmental effects would have minimal influences on the phenotype.

Our current results, however, suggest that targeting TGFα and its receptor might be beneficial in a subset of patients, potentially younger patients who have experienced a joint injury. It is possible that TGFα plays an important role post-trauma. Our previous *in vitro* studies showed that in addition to the loss of chondrocyte phenotype, TGFα had an effect on chondrocyte proliferation both in primary articular chondrocyte cell culture and in an articular cartilage organ culture system^25^. In the organ culture system, proliferation was evident based on the presence of chondrocyte clusters, a well-established sign of early OA, believed to represent an attempt at cartilage repair^25^,^48^. Thus it is possible that TGFα is produced locally within the joint in response to injury, but lacks the ability to successfully repair cartilage.

Overall it appears that TGFα, and more generally EGFR signaling, plays an important and complex role in post-traumatic OA progression. It appears that this signaling pathway can either protect from or enhance tissue destruction; but in both scenarios, the underlying mechanisms need to be studied further to examine whether this pathway is a suitable target for development of novel DMOADs. Moreover, protection of *Tgfa* null mice in one model of OA, but not in others in our study, further highlights the heterogeneity of this disease. These results also suggest that results from surgical models of OA, especially when done in young adult rodents, cannot necessarily be extrapolated to other forms of the disease.

## Methods

### Transforming growth factor alpha null mice

*Tgfa* null mice in a C57BL/6 genetic background were purchased from The Jackson Laboratory (Bar Harbor, Maine, USA)^49^. All animals were cared for and all experiments were carried out in accordance with the University of Western Ontario’s Animal Care and Use Guidelines. All animal protocols were approved by the Animal Use Subcommittee of the University Council on Animal Care. DNA from digested ear and/or tail clippings was used in PCR genotyping as described^47^. Two separate genotyping programs were used to amplify both the wild type *Tgfa* allele and the neo-cassette found in the mutated alleles. Oligonucleotides TGFalphaA (5′-GACTAGCCTGGGCTACACAGTG-3′) TGFalphaC (5′-ACATGCTGGCTTCTCTTCCTGC-3′), NeoForward (5′-CTTGGGTGGAGAGGCT ATTC-3′) and NeoReverse (5′-AGGTGAGATGACAGGAGATC-3′) were purchased from Sigma-Aldrich (Oakville, Ont., Canada) and all other genotyping reagents were purchased from Applied Biosystems Incorporated (Foster City, CA, USA). All direct comparisons were made between heterozygote and homozygote mutant siblings with at least four littermate pairs per time point per parameter examined.

### Destabilization of medial meniscus (DMM) model

To study OA in *Tgfa* mutant animals, we employed the well-established DMM model^27^. Our laboratory has expertise in employing this surgical technique^50^. In this model, the medial meniscotibial ligament (which anchors the medial meniscus to the tibial plateau) is cleaved, resulting in joint destabilization. DMM was performed on the left knee joint of ten-week old male mice as previously described^51^. Both *Tgfa* knockout mice and their heterozygous littermates were used in our studies. Heterozygous mice served as controls as previous studies have shown that *Tgfa* heterozygous mutants are phenotypically indistinguishable from their wild type littermates^49^. Sham surgeries consisting of an incision to the left knee were also performed on both genotypes. A veterinarian (I.W.) and veterinarian technician (T.H.) with previous experience in the DMM procedure performed all surgeries. Mice were housed individually after surgeries. At seven and fourteen weeks post-surgery, mice were sacrificed and their knee joints were isolated for histology.

To examine the effects of age and injury in combination, we also performed DMM surgeries as described above in six-month-old mice, and assessed disease progression at seven weeks post-surgery.

### Spontaneous osteoarthritis

To study the spontaneous development of OA, male and female *Tgfa* null mice were housed with their heterozygous littermates until eighteen months of age. Several male pairs were separated throughout this time course due to fighting. At eighteen months of age, mice were sacrificed and their knee, ankle and elbow joints were isolated and prepared for histology.

### Mouse tissue processing and histology

Tissues were fixed overnight in 4% PFA, and decalcified in 5% EDTA in PBS. Decalcification was determined by physical end-point test. Tissues were then processed, embedded in paraffin wax, and 5 μm thick serial sections were cut in the sagittal plane starting from the medial joint compartment. Previous studies have shown that the most severe damage in both DMM and spontaneous models of OA occur in the medial compartment^31^,^32^.

### Safranin-O/fast green staining

Sections were dewaxed in xylene and rehydrated through a series of graded ethanols ending in water. Tissues were then stained in 0.02% fast green for 25 minutes, dipped in 1% glacial acetic acid, then stained in 0.1% safranin-O for 7 minutes. Tissues were dehydrated and mounted using a xylene-based mounting medium.

### Immunohistochemistry

Immunohistochemistry was performed as previously described by our laboratory^47^,^52^,^53^. Primary antibodies against MMP3 (Abcam, San Francisco, CA), MMP13 (Abcam, San Francisco, CA) and type II collagen neoepitopes, exposed when type II collagen is cleaved by MMP13, were used^54^. Sections were first dewaxed in xylene, and rehydrated through a series of graded ethanol solutions ending in water. Antigen retrieval was performed in 10 mM sodium citrate at 95 °C for fifteen minutes. Sections were blocked in 5% goat serum, then incubated with primary antibodies overnight. Lastly, tissues were incubated in HRP-conjugated secondary antibody and visualized with the substrate DAB (brown precipitate). All tissues were visualized using a Leica DM Series inverted fluorescence/light microscope (Leica Microsystems, Richmond Hill, ON, Canada). At least three pairs of animals were stained for each antibody and multiple sections were used for each trial.

### OARSI scoring for DMM

For the DMM model, two blinded observers assigned a numerical grade and stage to each animal according to the Osteoarthritis Research Society International (OARSI) standards for OA histopathology^55^. This was the same scoring system used in our previous rat studies^24^,^56^. Five μm thick serial sections were cut in the sagittal plane of the knee joints, starting from the medial joint compartment. Approximately 5 sections of increasing depth were scored for each animal. One modification was made to the scoring system: a grade of 1 was assigned if the mouse cartilage had depleted safranin-O staining (representing depleted proteoglycan content) as this was determined to represent significant pathology, even in the absence of cartilage discontinuity. Scores were averaged for each group and statistical analysis was performed using a two-way ANOVA and Bonferroni post-tests.

### OARSI scoring for spontaneous OA

One blinded observer assigned a semi-quantitative score to each animal based on the OARSI recommendations for histopathological assessment of OA in the mouse^57^. Sections were collected and scored from the knee, elbow, and ankle joints as described above for DMM groups. Scores were averaged for each group and statistical analysis was performed using a paired t-test.

## Additional Information

**How to cite this article**: Usmani, S. E. *et al*. Context-specific protection of TGFα null mice from osteoarthritis. *Sci. Rep.* **6**, 30434; doi: 10.1038/srep30434 (2016).

## Supplementary Material

Supplementary Information
