# Supplementary material for: Context-specific protection of TGFα null mice from osteoarthritis
Source: Sci Rep. 2016 Jul 26;6:30434. doi: 10.1038/srep30434 (PMC4960644; doi:10.1038/srep30434)
Supplement: Supplementary Information [file srep30434-s1.pdf]

Supplementary Information

Context-specific protection of TGFα null mice from osteoarthritis

Shirine E. Usmani[1], Veronica Ulici[1], Michael A. Pest[1], Tracy L. Hill[2], Ian D. Welch[2], and Frank Beier[1]

[1]Department of Physiology & Pharmacology, Schulich School of Medicine & Dentistry, The University of Western Ontario, London, ON, Canada

[2]Department of Animal Care and Veterinary Services, University of Western Ontario, London, Canada

Corresponding Author: Frank Beier (email: fbeier@uwo.ca)

Key Words: transforming growth factor alpha, osteoarthritis, knockout mouse, destabilization of medial meniscus (DMM), OARSI score, histopathology

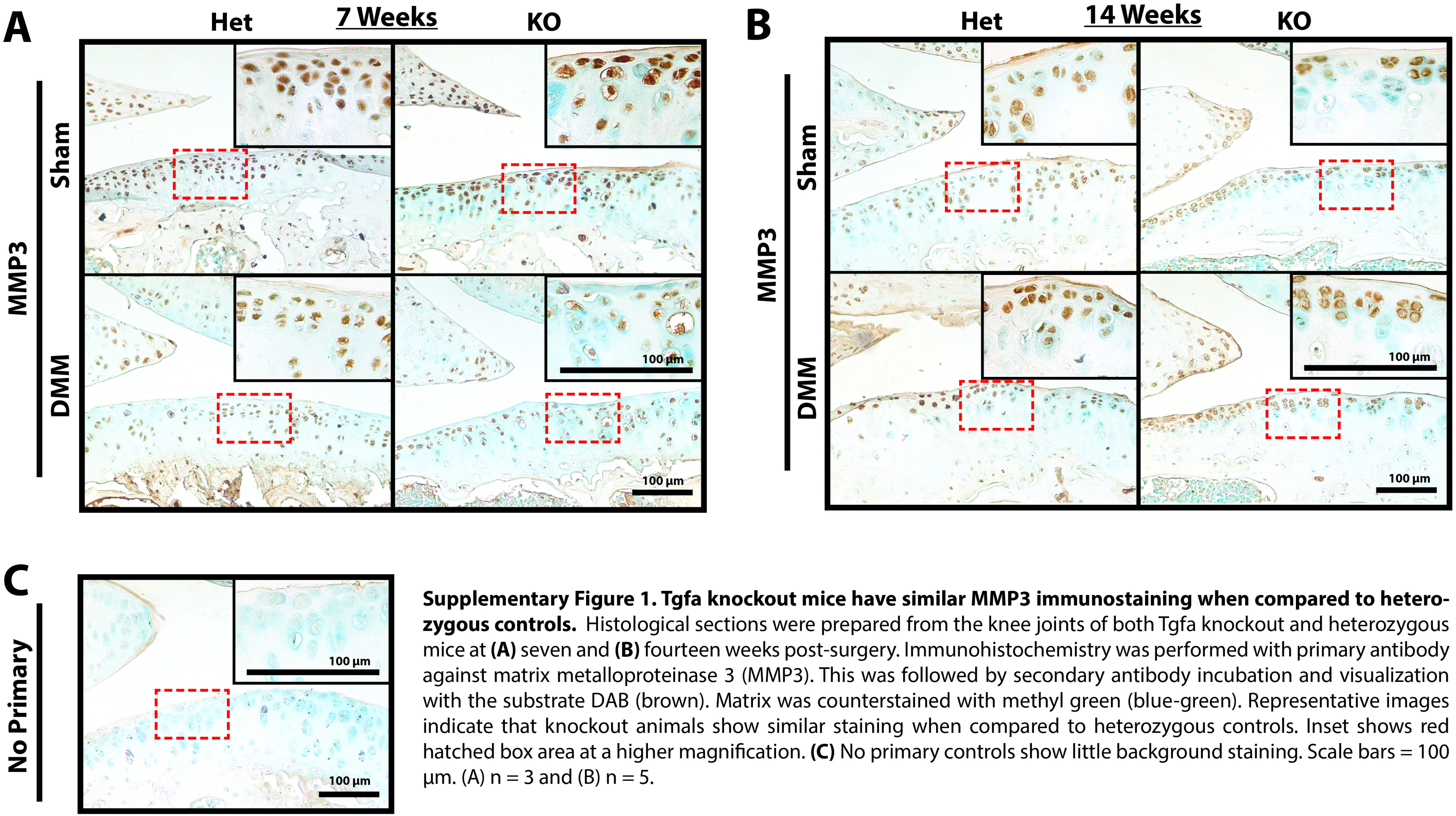

Supplementary Figure 1. Tgfa knockout mice have similar MMP3 immunostaining when compared to heterozygous controls. Histological sections were prepared from the knee joints of both Tgfa knockout and heterozygous mice at **(A)** seven and **(B)** fourteen weeks post-surgery. Immunohistochemistry was performed with primary antibody against matrix metalloproteinase 3 (MMP3). This was followed by secondary antibody incubation and visualization with the substrate DAB (brown). Matrix was counterstained with methyl green (blue-green). Representative images indicate that knockout animals show similar staining when compared to heterozygous controls. Inset shows red hatched box area at a higher magnification. **(C)** No primary controls show little background staining. Scale bars = 100 µm. (A) n = 3 and (B) n = 5.
